# Supplementary material for: The Association between Telomere Length and Head and Neck Cancer Risk: A Systematic Review and Meta-Analysis
Source: Int J Mol Sci. 2024 Aug 19;25(16):9000. doi: 10.3390/ijms25169000 (PMC11354702; doi:10.3390/ijms25169000)
Supplement: Supplementary file 1 [file ijms-25-09000-s001.zip › ijms-3094118-supplementary.pdf]

Table S1. **Sensitivity Analysis of HNC Risk [37–45].**

Excluded Study (Author, Year)	Summary OR and 95% CI without excluded study
Wu, 2003	1.29 (1.03-1.61)
Aida, 2010	1.32 (1.06-1.65)
Liu, 2011	1.48 (1.14-1.93)
Zhang OCC, 2013	1.35 (1.06-1.71)
Zhang OPSC, 2013	1.46 (1.15-1.85)
Total Zhang, 2013*	1.44 (1.11-1.85)
Bau, 2013	1.27 (1.02-1.57)
Gu OCC, 2016	1.45 (1.08-1.97)
Gu Other, 2016	1.48 (1.14-1.92)
Total Gu, 2016*	1.60 (1.09-2.35)
Paiva, 2018	1.30 (1.04-1.63)
Boscolo-Rizzo, 2020	1.39 (1.10-1.75)
Vaiciulis, 2020	1.45 (1.15-1.84)

Abbreviations: OR: Odds Ratios, **CI**: Confidence Interval, **HNC**: Head and Neck Cancer, **OCC**: Oral Cavity Cancer, **OPSC**: Oropharyngeal Squamous Cell Carcinoma. **Notes: *** The "Total Zhang, 2013" and "Total Gu, 2016” rows represent the combined exclusion of both Zhang and Gu datasets.
